# Asthma control using a combination of mometasone furoate/formoterol: grouped analysis of three clinical trials

**DOI:** 10.1186/1710-1492-7-S2-A16

**Published:** 2011-11-14

**Authors:** Martha White, Hendrik Nolte, Eli Meltzer, Robert Nathan

**Affiliations:** 1 Institute for Asthma & Allergy, Wheaton, MD, USA; 2 Merck Research Laboratories, Kenilworth, NJ, USA; 3 Allergy and Asthma Medical Group and Research Center, San Diego, CA, USA; 4 Asthma and Allergy Associates and Research Center, Colorado Springs, CO, USA

## Objective

We characterized the ability of mometasone furoate/formoterol (MF/F) combination to improve asthma control in adults/adolescents inadequately controlled on low-, medium-, and high-dose inhaled corticosteroids (ICS).

## Methods

Changes from baseline to endpoint (last evaluable visit) in Asthma Control Questionnaire (ACQ) scores were assessed in subjects from 3 Phase III trials (low- [n=746], medium- [n=781], and high-dose [n=728] previous ICS use). The ACQ categorizes asthma symptoms and use of rescue-medication using a 7-point scale (0=totally controlled, 6=severely uncontrolled). In two placebo controlled trials, subjects were randomized to receive MF/F (100/10μg or 200/10μg), MF (100μg or 200μg), F (10μg), or placebo (26 weeks; all twice-daily [BID] via metered-dose inhaler [MDI]); In a non-placebo controlled trial, subjects were randomized to receive MF/F 200/10μg, MF/F 400/10μg, or MF 400μg (12 weeks; all BID via MDI).

## Results

Baseline ACQ scores (1.23–1.38 (MF/F 100/10μg study), 1.41–1.47 (MF/F 200/10μg study), and 1.83–1.87 (MF/F 200/10 and 400/10μg study) indicated that subjects in all trials were not well controlled (<0.75) on ICS monotherapy. MF/F yielded ACQ improvements (100/10μg=–0.36; 200/10μg=–0.40) vs MF (100μg=–0.26; 200μg=–0.23), and deteriorations with F 10μg (+0.07; +0.11) and placebo (+0.24; +0.14). MF/F 400/10μg improved asthma control by –0.51 compared with –0.33 for MF 400μg monotherapy. Improvements for MF/F at all doses achieved minimal importance difference of ≥0.5 point increase.

## Conclusion

MF/F showed clinically important improvement in asthma control at all strengths and was better than MF, F and placebo.

